# The *sat1* Gene Is Required for the Growth and Virulence of the Human Pathogenic Fungus Aspergillus fumigatus

**DOI:** 10.1128/spectrum.01558-21

**Published:** 2022-02-02

**Authors:** Yunyun Wei, Dan He, Busi Zhao, Yuhuan Liu, Song Gao, Xiaowei Zhang, Li Wang

**Affiliations:** a Department of Pathogenobiology, Jilin University Mycology Research Center, Key Laboratory of Zoonosis Research, Ministry of Education, College of Basic Medical Sciences, Jilin University, Changchun, China; b Beijing ZhongKaiTianCheng Bio-technology Co. Ltd., Beijing, China; Universidade de Sao Paulo

**Keywords:** *Aspergillus fumigatus*, *sat1*, growth, virulence, cell wall, mitochondrion

## Abstract

Aspergillus fumigatus is an important opportunistic pathogenic fungus that causes invasive aspergillosis in immunocompromised humans. Regulated fungal growth is essential for disease development and progression. Thus, screening for genes that regulate fungal growth may lead to the identification of potential therapeutic targets for invasive aspergillosis (IA). Screening of the transfer DNA (T-DNA) random-insertion A. fumigatus mutants identified a severe growth deficiency mutant AFM2954 and featured *sat1* as the mutated gene described as a putative intracellular protein transporter of unknown function. The deletion of *sat1* exhibited severe growth defects and significantly increased the nematode and mouse survival rates and decreased the fungal loads and histopathological damages in mouse lungs. Transcriptomic analyses revealed expression changes associated with the cell wall synthesis, the tricarboxylic acid cycle (TCA cycle), and oxidative phosphorylation genes in the *sat1* mutant. Deletion of the gene resulted in resistance to cell wall-perturbing agents and thickened cell wall as well as reduced ATP contents and mitochondrial membrane potential, suggested that *sat1* affected the cell wall synthesis and mitochondrial function of A. fumigatus. All together, our study uncovered novel functions of *sat1* in growth and virulence of A. fumigatus and provided a theoretical basis for the development of new therapeutic target for treating IA patients.

**IMPORTANCE** Aspergillus fumigatus is the main causative agent of invasive aspergillosis in immunocompromised hosts, with up to 90% lethality. Nevertheless, the fungal factors that regulate the pathogenesis of A. fumigatus remain largely unknown. Better understanding of the mechanisms controlling growth of A. fumigatus may provide novel therapeutic targets. In the present study, we characterized *sat1* in the opportunistic pathogen A. fumigatus. The function of *sat1* remains unknown. We proved its important role in growth and virulence, likely because of its effects on cell wall synthesis and mitochondrial functions.

## INTRODUCTION

Aspergillus fumigatus is responsible for 90% of the reported cases of invasive aspergillosis (IA), which is the most common invasive infection caused by filamentous fungi (1–3). In recent years, the increasing number of immunocompromised individuals and the emergence of antifungal-resistant strains have resulted in a continuous increase in the incidence of IA, with mortality rates reaching 50% to 90% (4–6). In immunocompromised individuals, the inhaled conidia can germinate into hyphae, which can invade host tissues and blood vessels, eventually leading to IA (1, 7).

Studies suggest that there are some associations between virulence and the growth of microorganisms. For example, knocking out the mycobactin biosynthesis gene *mbtE* in Mycobacterium tuberculosis leads to severe growth defects and decreased virulence (8). In fungi, silencing genes related to calcineurin pathways, the cell wall integrity signaling pathway, and the tricarboxylic acid cycle (TCA cycle) adversely affect growth and virulence (9–11). Screening for regulatory genes that determine fungal growth may identify gene-associated virulence, thereby clarifying the mechanisms underlying fungal pathogenicity and revealing potential antifungal targets.

To search for genes regulating A. fumigatus growth and analyze their contribution to virulence, Agrobacterium tumefaciens-mediated transformation (ATMT) was used to construct a transfer DNA (T-DNA) random insertion mutation library (12). The mutant of AFM2954 with severe growth defect was obtained by screening the library for colonies with altered morphologies. The mutation in this strain was an insertion in *sat1*, which is a gene with unknown function. In this study, we functionally characterized A. fumigatus *sat1* regarding its effects on growth and virulence and analyzed the underlying molecular mechanisms.

## RESULTS

### Generation of *sat1* mutants and comparison of the corresponding genes in common pathogenic fungi.

**FIG 1:**
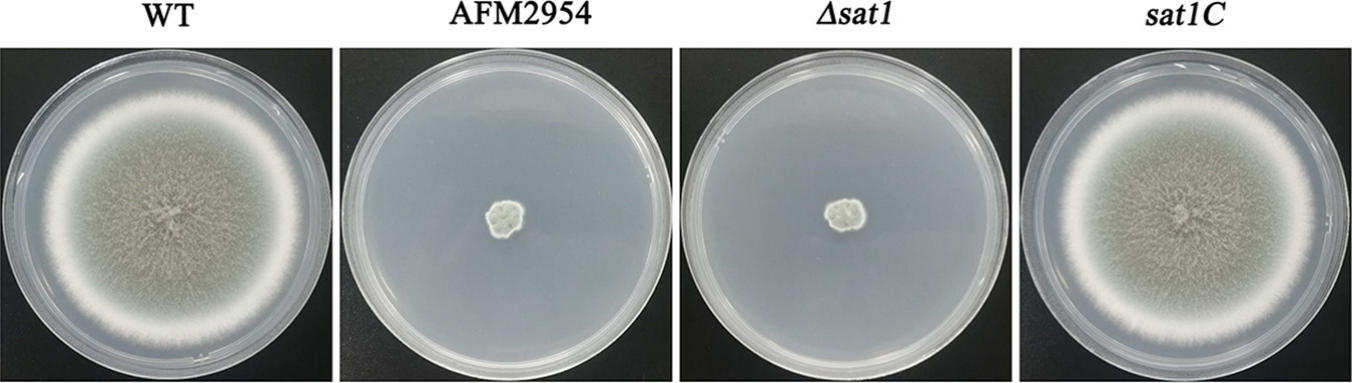
Disruption of *sat1* affected colony morphology of A. fumigatus. Strains were inoculated on PDA medium at 37°C for 3 days.

Wild-type (WT) A. fumigatus IFM40808 was used to construct a T-DNA random insertion mutant library by ATMT. A comparison of the colony morphologies of the mutants and the WT control revealed a mutant named AFM2954 exhibiting a severe growth deficiency (Fig. 1).

The T-DNA flanking sequence in AFM2954 was amplified by touchdown thermal asymmetric interlaced PCR (TAIL-PCR) (13). A BLAST search of the A. fumigatus Af293 whole genome indicated the T-DNA replaced a 28-bp segment in the first exon of AFUA_2G12380, which encodes the putative intracellular protein transporter *sat1*. To verify the gene identity and function, we constructed the targeted knockdown strain *Δsat1* and the complementation strain *sat1C*. The colony morphologies of these strains were similar to those of AFM2954 and WT, respectively (Fig. 1), indicating that the *Δsat1* and *sat1C* strains were successfully constructed.

**FIG 2:**
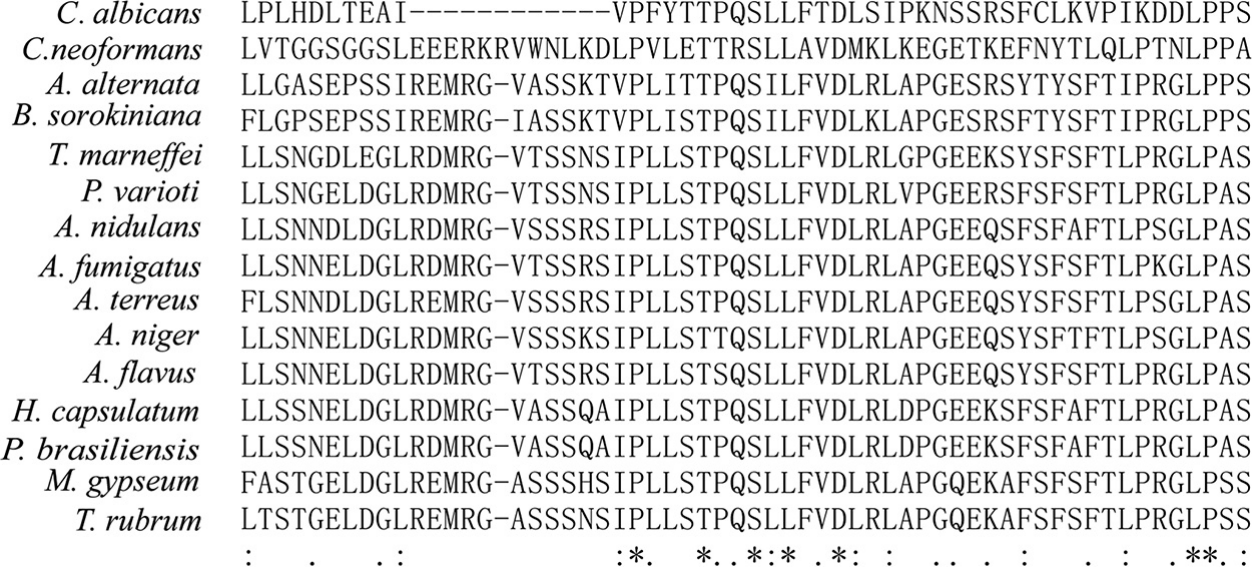
*sat1* is conserved among common pathogenic filamentous fungi. We performed a multiple-sequence alignment of *sat1*-like proteins using MUSCLE (https://www.ebi.ac.uk/Tools/msa/muscle/), and we showed a partial of the alignment result. Candida albicans, GenPept accession no. XP_721015.2; Cryptococcus neoformans, GenPept accession no. XP_777267.1; Alternaria alternate, GenPept accession no. XP_018390575.1; Bipolaris sorokiniana, GenPept accession no. XP_007694569.1; Talaromyces marneffei, GenPept accession no. XP_002148790.1; Paecilomyces varioti, GenPept accession no. XP_028487758.1; Aspergillus nidulans, GenPept accession no. XP_663893.1; Aspergillus fumigatus, GenPept accession no. XP_755570.1; Aspergillus terreus, GenPept accession no. XP_001208535.1; Aspergillus niger, GenPept accession no. XP_025460961.1; Aspergillus flavus, GenPept accession no. KAF7620608.1; Histoplasma capsulatum, GenPept accession no. XP_001541714.1; Paracoccidioides brasiliensis, GenPept accession no. XP_010762527.1; Microsporum gypseum, GenPept accession no. XP_003174688.1; Trichophyton rubrum, GenPept accession no. XP_003236425.1.

The A. fumigatus *sat1* gene is located on chromosome 2 and comprises 2,608 bases, with 3 exons and 2 introns. The encoded protein contains 831 amino acids, but its function remains unknown. An amino acid sequence analysis indicated similar proteins are produced by other fungal species (Fig. 2). More specifically, the A. fumigatus *sat1* amino acid sequence was revealed to be most similar to homologous proteins in other common Aspergillus species (67% to 72%) and less similar to homologous proteins in other filamentous fungi (40% to 62%). It does not share homology with Cryptococcus neoformans and Candida albicans (11% to 17% amino acid identity).

### Phenotypic analysis of *sat1* mutant.

**FIG 3:**
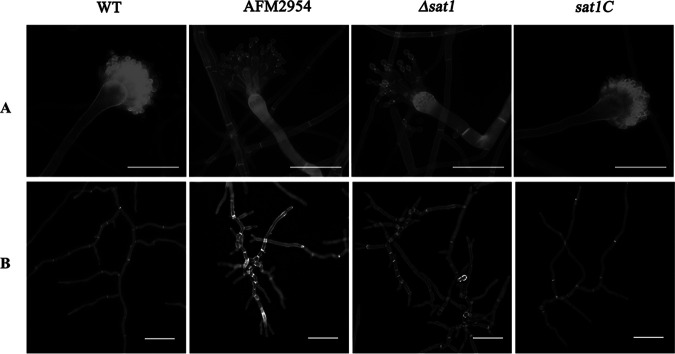
Disruption of *sat1* affected the micromorphology of A. fumigatus. (A) Phenotypes of conidial head. Strains were inoculated on PDA for 24 h. Bar, 50 μm. (B) Hyphal morphology. Strains were inoculated in PDB for 12 h. Bar, 50 μm. They were observed by Fluorescent Brightener 28 staining (35 μg/mL).

The AFM2954 and *Δsat1* colonies grew very slowly and had a raised, wrinkled appearance (Fig. 1). Additionally, the AFM2954 and *Δsat1* strains produced abnormally shaped conidial heads, with smaller vesicles, fewer phialides, and more phialide layers than the WT conidial heads. Moreover, the phialides in the AFM2954 and *Δsat1* strains were elongated and had enlarged apices (Fig. 3A). The AFM2954 and *Δsat1* strains exhibited aberrant mycelial morphology, which was reflected by the swelling and branching of the hyphal tip as well as increased separation (Fig. 3B).

### Disruption of *sat1* decreased A. fumigatus virulence.

**FIG 4:**
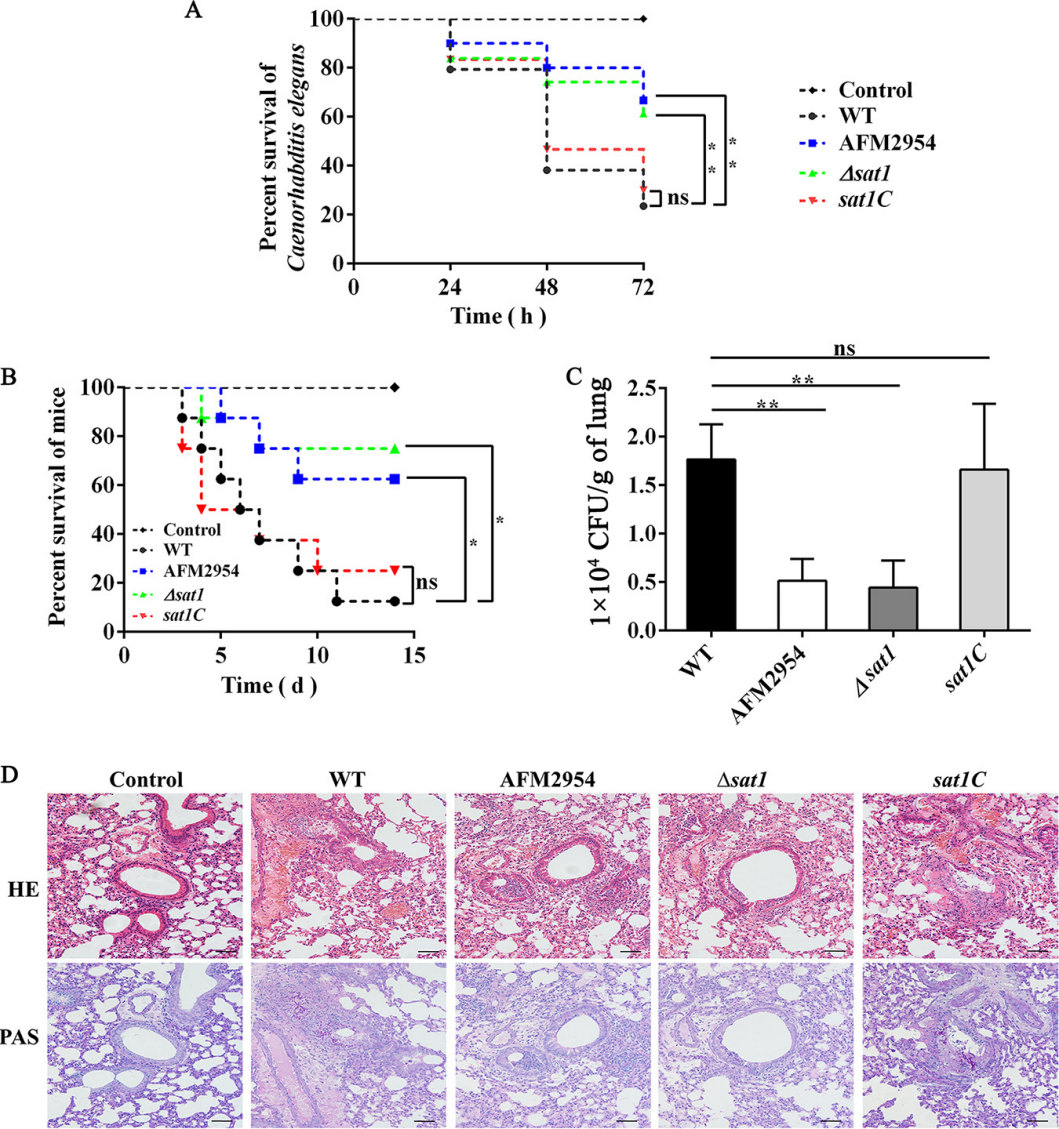
Deletion of *sat1* significantly reduced the virulence of A. fumigatus. (A) Loss of the *sat1* gene significantly increased the survival rates of the infected Caenorhabditis elegans. The nematodes were infected by 100 μL of 2.0 × 10^8^ CFU spore suspension. Survival rates over a 72-h observation period are shown. (B) Loss of the *sat1* gene significantly increased the survival rates of the infected mice. We challenged BALB/c mice with 30 μL 6 × 10^6^ CFU spore suspension via intranasal instillation. The survival rates over a 14-day postinfection period. On day 3 after infection, a pulmonary lobe of each mice for fungal burden (C), and the other was used for histopathologic analysis (D). Lung tissue sections were stained with hematoxylin and eosin (H&E) and periodic acid-Schiff stain (PAS), respectively. Bar, 200 μm. *, *P < *0.05; **, *P < *0.01; ns, not significant.

We evaluated the effect of *sat1* on virulence using the Caenorhabditis elegans model, which has no adaptive immune system or specialized immune cells (14, 15). The lack of host immune system effects on fungi was conducive to assessing the virulence of the strains. The survival rates of the nematodes infected with the WT and *sat1C* strains were 21% and 30%, respectively, whereas the survival rates of the nematodes infected with the AFM2954 and *Δsat1* strains were 67% (*P = *0.006) and 61% (*P = *0.007), respectively (Fig. 4A). A similar trend was observed in the mouse model. The survival rates were higher for the mice infected with the AFM2954 (62%, *P = *0.0250) and *Δsat1* (75%, *P = *0.0178) strains than for the mice infected with the WT (12.5%) and *sat1C* (25%) strains (Fig. 4B). Moreover, the disruption of *sat1* decreased the fungal load in mouse lungs (Fig. 4C), which had intact bronchial walls and a small number of hyphae. In contrast, the infections by the WT and *sat1C* strains resulted in mouse lungs with disrupted bronchial walls, a substantial abundance of red blood cells, and a relatively large number of hyphae (Fig. 4D). These results suggest that *sat1* is important for virulence of A. fumigatus.

### RNA-seq analyses.

**FIG 5:**
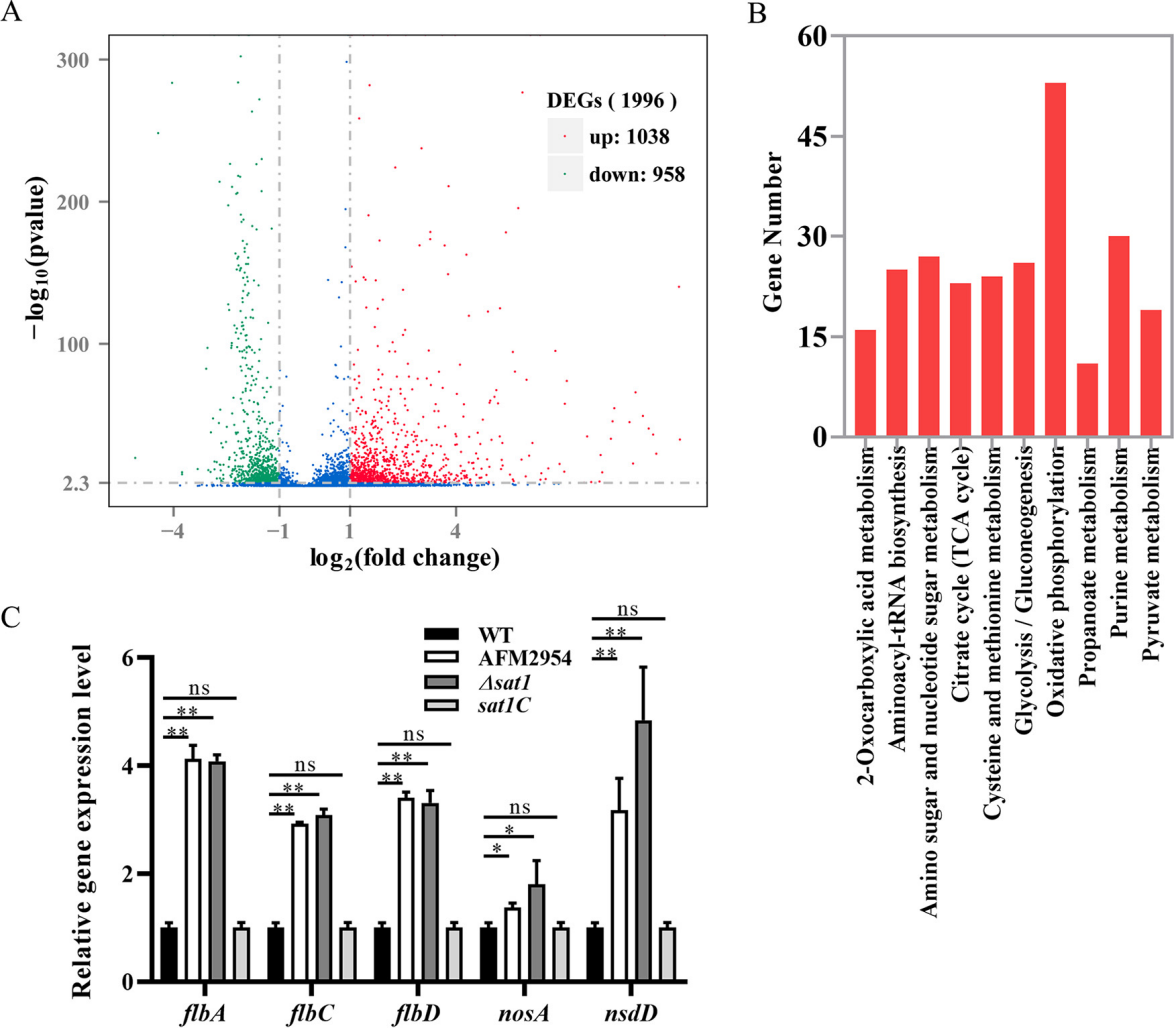
Genome-wide transcriptome analysis of WT and AFM2954 strains. (A) Volcano plot of differentially expressed genes (DEGs). (B) Pathway enrichment of DEGs. Only the top 10 enriched pathway terms are presented. (C) Relative expression levels of development genes, which were obtained by qRT-PCR and normalized against the 18S rRNA gene. *, *P < *0.05; **, *P < *0.01; ns, not significant.

To investigate the possible mechanism of the roles of *sat1* on growth and virulence of A. fumigatus, transcriptome (RNA-seq) analysis was performed; 1,996 genes showed differential expression, of which 1,038 were upregulated and 958 were downregulated (Fig. 5A). KEGG pathway enrichment analysis showed that the differentially expressed genes (DEGs) were significantly enriched in metabolic pathways, including oxidative phosphorylation (OXPHOS), TCA cycle, amino sugar and nucleotide sugar metabolism, and purine metabolism; among these, the enrichment of OXPHOS and TCA cycle were most significant (Fig. 5B).

We observed the effects of *sat1* gene deletion on the development of A. fumigatus (Fig. 3). Spore development-related genes, including *flbA*, *flbC*, *flbD*, *nosA*, and *nsdD* (16), were upregulated in the RNA-seq data (Table S1 in the supplemental material), which was also validated by reverse transcription-quantitative PCR (qRT-PCR) (Fig. 5C). The results were consistent with the RNA-seq analysis, which indicates that the date was reliable.

### Disruption of *sat1* affected A. fumigatus mitochondrial functions.

**FIG 6:**
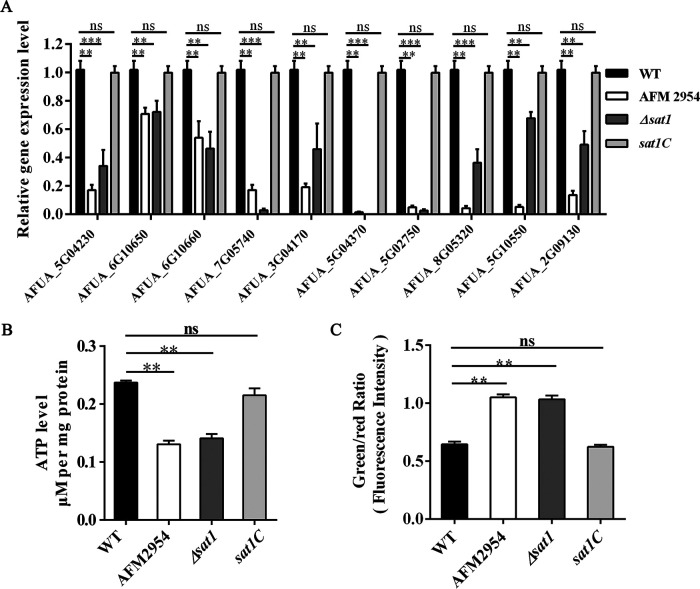
Disruption of *sat1* affected mitochondrial function of A. fumigatus. (A) Relative expression levels of TCA cycle and OXPHOS genes, which were obtained by qRT-PCR and normalized against the 18S rRNA gene. (B) Loss of the *sat1* gene reduced intracellular ATP production. (C) Loss of the *sat1* gene decreased mitochondrial membrane potential, determined by the ratio of green and red fluorescence using JC-1. **, *P < *0.01; ***, *P < *0.001; ns, not significant.

In the RNA-seq data, *sat1* knockdown resulted in the downregulation of 21 TCA cycle genes and 52 OXPHOS genes (Table S1). We randomly selected 10 genes related to the TCA cycle and OXPHOS for a qRT-PCR analysis. All 10 genes were expressed at lower levels in the AFM2954 and *Δsat1* strains than in the WT and *sat1C* strains (Fig. 6A). The qRT-PCR results were largely consistent with the RNA-seq data.

The TCA cycle generates electron donors which drive the production of ATP via mitochondrial OXPHOS. We detected the levels of ATP in A. fumigatus cells. Compared with WT and *sat1C*, the ATP concentration of AFM2954 and *Δsat1* cells was lower (Fig. 6B). The mitochondrial membrane potential (MMP) serves as a surrogate for OXPHOS rates; thus, we examined the MMP. The ratio of green (JC-1 monomer) to red (JC-1 aggregate) fluorescence was higher in AFM2954 and *Δsat1* than in WT and *sat1C* (Fig. 6C), indicating reduced MMP in the former two strains. Taken together, these results indicated that the disruption of *sat1* adversely affected A. fumigatus mitochondrial functions.

### *sat1* is involved in cell wall synthesis-related processes.

**FIG 7:**
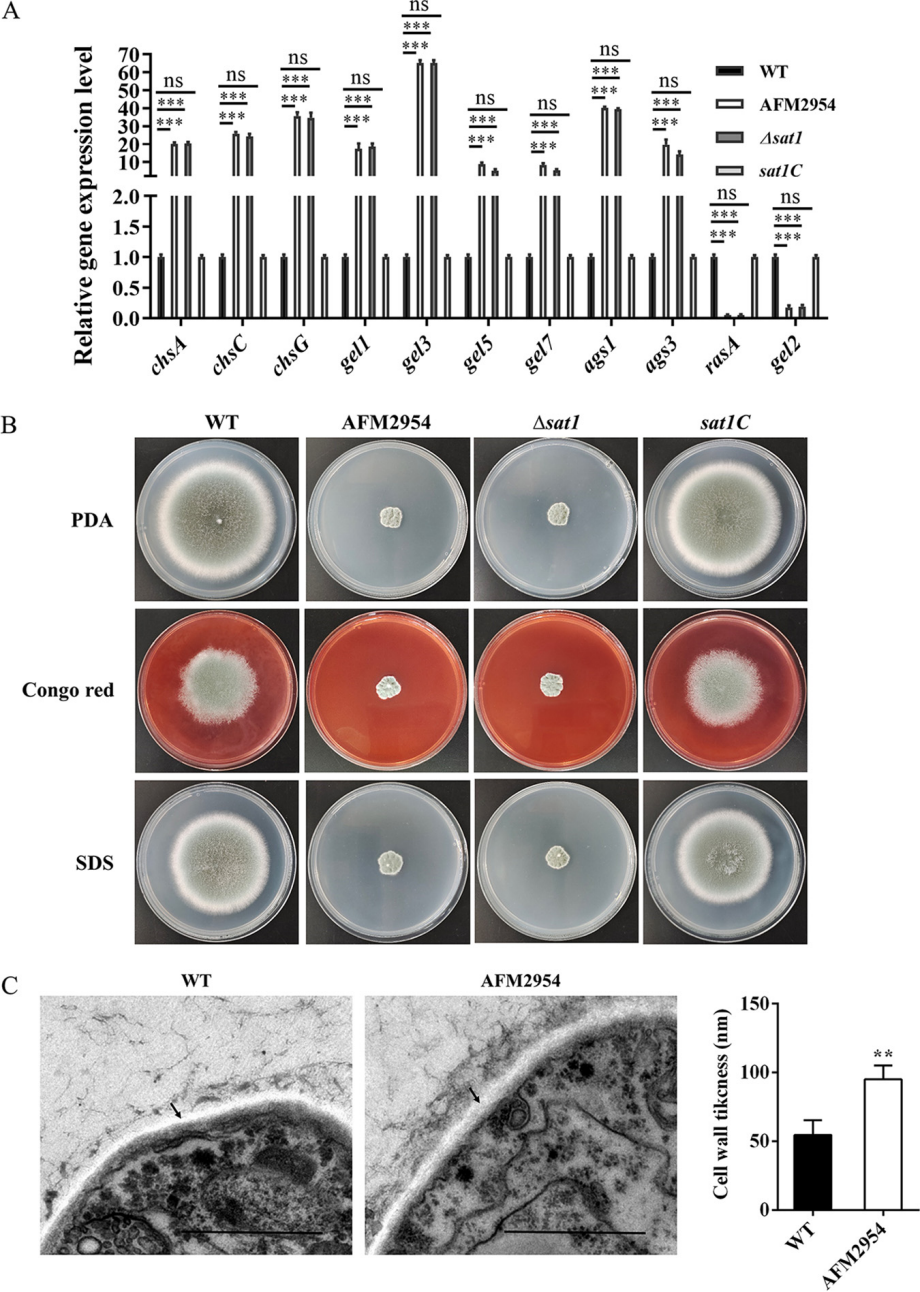
Deletion of *sat1* enhances resistance to cell wall-perturbing agent and increased wall thickening of A. fumigatus. (A) Relative expression levels of cell wall synthesis genes, which were obtained by qRT-PCR and normalized against the 18S rRNA gene. (B) Deletion strains were resistant to cell wall-damaging agents. Two microliters of 1 × 10^6^ CFU spore suspension were grown on PDA medium supplemented with 200 μg/mL Congo red (CR) or 0.02% SDS at 37°C for 3 days. (C) Loss of the *sat1* gene significantly increased cell wall thickness. Transmission electron microscopy (TEM) of hyphal sections of the A. fumigatus WT and AFM2954 strains were grown for 24 h in PDA at 37°C. Bar, 1 μm. The cell wall thickness was measured using ImageJ software. Length of three random points on the cell wall was measured, and the average of these values was the result. **, *P < *0.01; ***, *P < *0.001; ns, not significant.

In addition to altered metabolic pathways, we also found that many genes involved in the synthesis of the cell wall had altered expression in AFM2954 compared to WT, such as chitin synthase (A, C, and G) and 1,3-β-glucanosyltransferase (Gel1, Gel3, Ge5, and Gel7) being upregulated and Gel2 and RasA downregulated, which were consistent with those of qRT-PCR (Fig. 7A). Next, we further investigated whether it also responded to cell wall stress. The WT and *sat1C* strains exhibited significant growth inhibition on potato-dextrose agar (PDA) medium supplemented with 200 μg/mL Congo red (CR) or 0.02% SDS. In contrast, AFM2954 and *Δsat1* strains seemed minimally unaffected, which indicates that *sat1* knockdown increased resistance to cell wall-perturbing agents (Fig. 7B). Transmission electron microscopy (TEM) showed that the cell wall of the AFM2954 strain was thicker than that in the WT (Fig. 7C). These results indicate that *sat1* plays a role in cell wall synthesis.

## DISCUSSION

In this study, the ATMT-based random insertion of T-DNA resulted in the identification of the *sat1* gene as a regulator of A. fumigatus growth. An amino acid sequence analysis indicated that *sat1* is conserved among multiple pathogenic filamentous fungi (Fig. 2); however, this protein has not been characterized, and its function is unknown. In the current study, we observed that disrupting *sat1* leads to severe colony growth defects (Fig. 1) as well as abnormal conidial heads and hyphal morphology (Fig. 3). These findings suggest that *sat1* is important for A. fumigatus growth.

Several studies proved that the virulence of mutant fungi with altered morphology (e.g., colony appearance and mycelial morphology) may be abnormal (17–22). The contribution of *sat1* to A. fumigatus virulence was initially evaluated using the C. elegans infection model. The deletion of *sat1* significantly increased the C. elegans survival rate (Fig. 4A), indicating this gene contributes to A. fumigatus virulence. To validate this finding, we performed experiments using a mouse model. Consistent with the C. elegans infection model, the mortality rate, fungal load, and lesion severity decreased in the mice infected with the *sat1*-disrupted strains (Fig. 4B to D). Collectively, these results suggest that deleting *sat1* decreases A. fumigatus virulence. These findings indicate that *sat1* encodes an important A. fumigatus virulence factor.

Calcineurin and cell wall integrity pathways, as well as mitochondria, are essential for fungal growth and virulence (23–25). We analyzed gene expression levels in AFM2954. The RNA-seq data revealed that the differentially expressed genes are involved in cell wall synthesis (Table S1 in the supplemental material). In the course of our experiments, we found that the preparation of protoplasts from *sat1* deletion strains requires a longer time through cell wall-degrading enzyme digestion. The *sat1* deletion strains showed increased resistance to cell wall-disturbing agents (Fig. 7B). We observed that increased cell wall thickness in the AFM2954 strain (Fig. 7C) may be the reason for the resistance to cell wall-disturbing agents, which might be related to the higher expression of chitin synthase and α-1,3-glucan synthase. Moreover, we found that genes encoding small monomeric GTPase RasA and β-1,3-glucanosyltransferase Gel2 were downregulated and α-1,3-glucan synthase Ags3 was upregulated in the *sat1* deletion strain compared to WT (Fig. 7A). Reports have suggested that deletion of *rasA* or *gel2* reduced growth and virulence in A. fumigatus (26, 27). Deletion of *ags3* enhanced the virulence in A. fumigatus (28). Therefore, the altered cell wall synthase may contribute to the growth defect and reduced virulence in the *sat1* deletion strains.

The TCA cycle generates electron donors, which drive the production of ATP via mitochondrial OXPHOS to generate 95% of the energy needed by eukaryotic cells (29, 30). The RNA-seq data revealed that the expression levels of genes involved in the TCA cycle and OXPHOS were downregulated in *sat1* deletion strain AFM2954. The decreased ATP contents and MMP in *sat1* deletion strains (Fig. 6B and C) suggested that *sat1* can affect A. fumigatus mitochondrial functions. A previous study demonstrated that knocking out key TCA cycle genes in C. albicans leads to decreased ATP production as well as defective growth (11). The inhibition of the respiratory chain affects the yeast-to-hyphae morphological transition and virulence of C. albicans (31). Therefore, *sat1* may affect energy supply to A. fumigatus by regulating mitochondrial functions and thereby influences hyphal growth.

In recent years, studies suggest that there are some associations between mitochondrial function, cell wall synthesis, and virulence in fungi. A role has been described for mitochondria in “masking” the β-glucan on the C. albicans cell surface, which results in avoidance of recognition by immune cells (32). Moreover, such as the C. albicans MMS2, the novel J-domain protein Mrj1 of C. neoformans, and the A. fumigatus Ssd1 are important for the mitochondrial functions, cell wall synthesis, and virulence of pathogenic fungi (31, 33–35). In the current study, we determined that disrupting *sat1* affects cell wall synthesis and negatively affects A. fumigatus mitochondrial functions and virulence.

In summary, we identified and characterized the novel function of *sat1*, a putative protein in A. fumigatus. The *sat1* might regulate the expression levels of TCA cycle and OXPHOS genes, thereby impacting mitochondrial membrane potential and the production of ATP, and *sat1* affected susceptibility to cell wall-disturbing agents and the cell wall thickness of A. fumigatus by regulating the expression levels of cell wall synthesis genes. Further, *sat1* affected growth and virulence of A. fumigatus. Hence, *sat1* may be a novel therapeutic target for treating IA patients.

## MATERIALS AND METHODS

### Strains, cells, and plasmids.

The WT A. fumigatus strain IFM40808, the T-DNA random insertion A. fumigatus mutants, Agrobacterium tumefaciens Agr0, and the pPTRII and pXEH plasmids were stored at the Jilin University Mycology Research Center (Jilin, China).

### Construction of the random insertion mutant library and analysis of the T-DNA insertion sites.

We constructed the T-DNA insertion mutant library according to a previously described method involving ATMT (12). The T-DNA flanking sequences were identified by touchdown TAIL-PCR (13), and the amplified fragments were sequenced by Comate Bioscience Co., Ltd. (Jilin, China). To determine the insertion sites, the resulting sequences were aligned with the A. fumigatus whole-genome sequence (NCBI taxonomy browser ID 330879) using the Basic Local Alignment Search Tool (BLAST) available on the NCBI website (http://blast.ncbi.nlm.nih.gov/).

### Construction of the *sat1* deletion strain and the complementation strain.

The method used for the targeted knockout of *sat1* was based on homologous genetic recombination by ATMT, with the hygromycin gene in the pXEH plasmid replacing the target gene.

To avoid problems associated with multiple gene copies, the complementation plasmid was derived from the pPTRII plasmid (36), which can replicate autonomously and is not integrated into the host genome. Additionally, *sat1* expression was under the control of the Aspergillus nidulans trpC promoter (37). We amplified the WT A. fumigatus *sat1* expression cassette. The pPTRII plasmid was digested with the restriction endonuclease HindIII. The three DNA fragments were ligated using the One Step Cloning kit (Vazyme, China). We subsequently performed polyethylene glycol (PEG)-mediated protoplast transformation to insert the recombinant plasmid into the *Δsat1* strain to generate the complementation strain *sat1C*. Details regarding the relevant primers are provided in Table S2 in the supplemental material.

### Growth analysis and morphological examination.

To examine the morphology of the conidial heads, PDA medium was inoculated with spores and incubated at 37°C for 24 h. The samples were then stained with Fluorescent Brightener 28 (Sigma, USA) and observed using the BX53 fluorescence microscope (Olympus, Japan). Additionally, potato dextrose broth (PDB) medium was inoculated with spores (1 × 10^5^ CFU) and incubated at 37°C for 12 h. The samples were then stained with Fluorescent Brightener 28 and observed using the IX71 fluorescence microscope (Olympus). The samples for transmission electron microscopy (TEM) were prepared following the method described by Weichert et al. (38). ImageJ was used to measure the thickness of cell wall. The length of three random points on the cell wall was measured, and the average of these values was calculated as a result of cell wall thickness (39).

### Animal infection models.

C. elegans infection models, which are relatively simple and cost-effective, have been widely used to evaluate fungal virulence (40–42). In this study, a C. elegans infection model was established as previously described (15). More specifically, the nematodes were infected by adding a 100 μL A. fumigatus spore suspension (2.0 × 10^8^ CFU).

A mouse infection model was developed as described by Zhou et al. (43). Briefly, male BALB/c mice were immunosuppressed with cyclophosphamide (150 mg/kg) and hydrocortisone (40 mg/kg) and infected intranasally with a 30 µL conidial suspension (6 × 10^6^ CFU). Mice were killed 3 days postinfection, and their tissues were harvested for analyses of the fungal load and histopathological characteristics. The survival rates of eight mice in each group were calculated for 14 days postinfection.

GraphPad Prism 6 (GraphPad Software, USA) was used to plot the survival curves and perform the log-rank test and the Wilcoxon test with Gehan-Breslow weighting to analyze the data.

### Fungal burden and histopathology assays.

Fungal burden and histopathology assays were performed as described previously (44, 45). Briefly, one side of the mice lungs was homogenized in phosphate-buffered saline (PBS) and using 10-fold gradient dilution for culture. The fungal burden was calculated by counting CFU. The other side of the lungs was embedded with paraffin, cut into slices, and stained with hematoxylin and eosin (H&E) or periodic acid-Schiff (PAS) for histopathologic analysis.

### RNA-seq and qRT-PCR analysis.

Spores (1 × 10^6^ CFU) were added to PDB medium and incubated at 37°C for 24 h. The resulting mycelia were collected and ground to a powder in liquid nitrogen. Total RNA was extracted from the ground material using RNAiso Plus (TaKaRa, Japan). The RNA served as the template for constructing cDNA libraries as described by Dong et al. (46). The libraries were sequenced using the HiSeq 2500 platform (Illumina, USA) by Novogene Corporation (Beijing, China). Gene expression levels were determined on the basis of the number of fragments per kilobase per million reads (FPKM). Genes were considered to be significantly differentially expressed if the following two criteria were satisfied: *P *< 0.005 and |log_2_ (fold change)| > 1. Differentially expressed genes enrichment analyses were performed using Kyoto Encyclopedia of Genes and Genomes (KEGG) databases.

The RTIII Super Mix (Monad, China) was used to generate cDNA for a qRT-PCR analysis, which was performed using the SYBR Green master mix (Monad) and the ABI QuantStudio 3 PCR system (Applied Biosystems, USA). The relative expression levels of genes related to the TCA cycle and oxidative phosphorylation (OXPHOS) were calculated using the threshold cycle (2^−ΔΔ^*^CT^*) method. The A. fumigatus gene expression levels were normalized against the expression of the 18S rRNA housekeeping gene. Details regarding the relevant primers are provided in Table S3.

### Mitochondrial function analysis.

To quantitatively analyze the intracellular ATP, 50 mg mycelia were collected and ground to a powder in liquid nitrogen. The ATP content was measured using the ATP assay kit (Beyotime, China). Luminescence was quantified using the Synergy H1 multidetection microplate reader (BioTek, USA) at wavelengths between 520 and 620 nm. The ATP content was calculated on the basis of a standard curve and was normalized against the protein concentrations.

The MMP was analyzed using the JC-1 mitochondrial membrane potential detection kit (Beyotime, China). Mycelia were collected by centrifugation, resuspended in the JC-1 working solution, and incubated at 37°C for 30 min. The mycelia were then washed and analyzed for fluorescence, which was quantified according to the manufacturer’s instructions.

### Data analysis.

Each experiment was replicated independently at least three times. Data are presented herein as the mean ± standard error unless otherwise indicated. A one-way analysis of variance was used to assess the differences in transcript levels. The significance of the differences between two groups was determined by Student's *t* test (α = 0.05).

### Ethics approval.

The study was conducted according to the Guidelines for Care and Use of Laboratory Animals of Jilin University, and approved by the Animal Ethics Committee of Jilin University (protocol code, 2021-139; approval date, 21 July 2021).
