# Supplementary material for: The sat1 Gene Is Required for the Growth and Virulence of the Human Pathogenic Fungus Aspergillus fumigatus
Source: Microbiol Spectr. 2022 Feb 2;10(1):e01558-21. doi: 10.1128/spectrum.01558-21 (PMC8809347; doi:10.1128/spectrum.01558-21)
Supplement: SUPPLEMENTAL FILE 2 — Supplemental material. Download SPECTRUM01558-21_Supp_1_seq11.pdf, PDF file, 0.4 MB [file spectrum01558-21_supp_1_seq11.pdf]

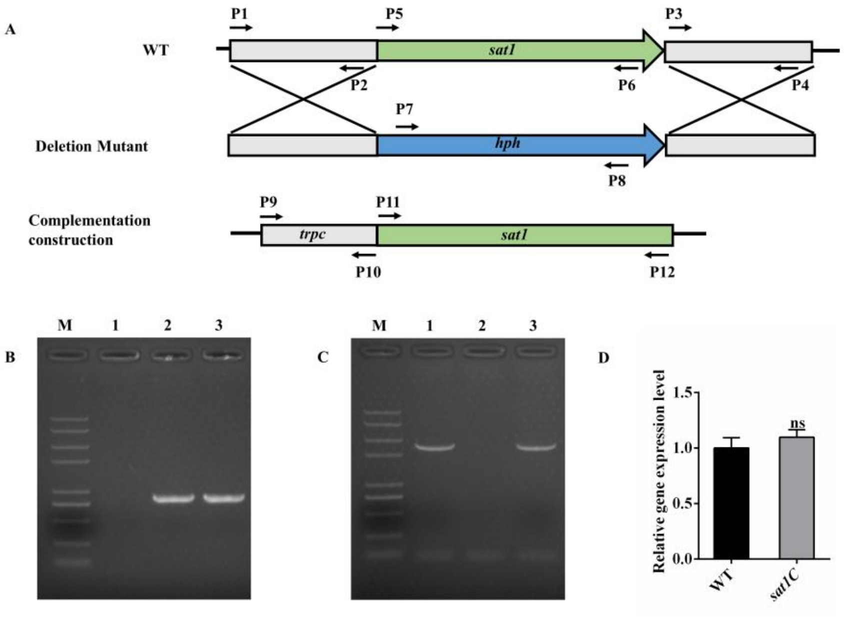

Supplementary Figure 1. Deletion and complementation of *sat1* of *A. fumigatus*. (A) Schematic diagram of WT, Δ*sat1* and *sat1C*. (B) The *hph* gene can be amplified from Δ*sat1* and *sat1C*, but cannot be amplified from WT. (C) *sat1* gene can be amplified from WT and *sat1C*, which cannot be amplified from Δ*sat1*. M: DNA molecular size marker Trans 2K plus II; 1: WT; 2: Δ*sat1*; 3: *sat1C*. (D) The mRNA level of *sat1* in WT and *sat1C*, which were determined by qRT-PCR.

Supplementary Table 2. Primers used in study for the deletion strain complementation strain

Primer Name	Nucleotide Sequence(5’ to 3’)	Purpose
P1	GGAAGATCTGAGGTCCTCGACAGTTCAGCTCGCT	Amplify sequence upstream of *sat1*
P2	CGGGGTACCGTCATATGCTAAACGAAGCAGGCCAT	
P3	CGCGGATCCCGACTGGCACCAGGAGAAGAACAGT	Amplify sequence downstream of

P4	CCCAAGCTT CAATACGAGTATCATAAGAAGACTCT	*sat1*
P5	ATGCAATCGGACATACAGGTCT	Amplify sequence of *sat1*
P6	CTAGATAGAATAACCCTCGTTCTCT	
P7	CGCCCAAGCTGCATCATCGAA	Amplify sequence of *hph*
P8	CGACAGCGTCTCCGACCTGA	
P9	GACCATGATTACGCCAAGCTTCAG-AAGATGATATTGAAGGAGCATTT	Amplify sequence of Trpc promoter
P10	CCGATTGCATATCGATATCGATGCTTCGGTAGA	
P11	CGATATCGATATGCAATCGGACATACAGGTCTT	Amplify sequence of *sat1* (for complementation)
P12	GACCTGCAGGCATGCAAGCTTCTAG-ATAGAATAACCCTCGTTCTCTTCC	

Supplementary Table 3. Primers used in study for qRT-PCR.

Primer Name	Nucleotide Sequence(5' to 3')	Purpose
18Sf1	CGGCCCTTAAATAGCC CGGTC	normalization
18Sr1	ACTCCCCTGAGCCAGTCCG	
Btstqf1	CAAGGATGCTGTCCAACCCC	AFUA_7G05740
Btstqr1	AATCACCGGCCTTCTTCTGC	
Atpaf1	GCAAACAGGGTCAATGGCTG	AFUA_6G10650
Atpar1	ACCCCAGTACATCTTGCTAACG	
Citf1	TGTTTGGGAGGGCTCTGTTC	AFUA_5G04230

Citr1	GGGATCTCACCAGTCAGCAG	
PdbAf1	GGACTGCACCCTATCTGCG	AFUA_3G04170
PdbAr1	AATCTTGGGAGTGCTGAGCG	
Atpbf1	AATTCCGTGCGAGAGGGTG	AFUA_6G10660
Atpbr1	TCTCCTCGTTGGAGGGGTAG	
Nadhf1	TCCATTGCTCGGAGTTCCC	AFUA_5G04370
Nadhr1	TACAGAGCCTGGGGAGACAT	
Cytf1	CCCACGAGGATGAGACATACG	AFUA_5G02750
Cytr1	CAGTTGTTGAGGTTACGCTGG	
Fatpf1	AAGCGTACTGGAGAGATTGTCG	AFUA_8G05320
Fatpr1	GTCTGGACAGGCTGGTTGAC	
Atpff1	AGGTCTCTCAACACTTGGGTG	AFUA_5G10550
Atpfr1	GGGGATCTTGATGGGAGCAC	
Matpf1	CTTTGCACCACGACACCCTG	AFUA_2G09130
Matpr1	AGGCCGTCTTCTGTTGTGTG	
FAf1	TTGTCGTCTTCCAGCCATCC	*flbA*
FAr1	ACGGAATAGCAGTCGCAGAG	
FCf1	AGCCAGGATTTGACACCTCG	*flbC*
FCr1	ATGGATGACCCGGACGAATG	
FDf1	AGAACCTGAAGCCCTCGTTG	*flbD*
FDr1	GGTTCATGCTGCCATTCCAC	

NAf1	TCATCTTCAGCATCGTCGGG	*nosA*
NAr1	CAGCCATTCCGCTGCATTAC	
NDf1	CCCATCCTCCACATGCTCTC	*nsdD*
NDr1	ATAGATGCGAGGCTTGACCG	
Ags3f1	GGCCTGGTCCCACTGTTAC	Ags3
Ags3r1	ACGGTTTCTCCCGTGTCTTC	
chAf1	GAGCAGCCAACTTACTCGGT	*chsA*
chAr1	ACAGCATAATCGTAGGCGGG	
Gel1f1	ACTGGCTACGGTCTTCCTCT	*gel1*
Gel1r1	CCGCTAATCTCCACCAGACC	
Gel3f1	ACTTCGAGACGACCAACGAC	*gel3*
Gel3r1	CCAGGTGTCATCGACGGTAG	
Ags1f1	TAGCATGTGGAAGTGGCAGG	*ags1*
Ags1r1	GCCACTGCTAGTCTTGGGTT	
ChsGf1	GCCAAGACCACCAAGGATGA	*chsG*
ChsGr1	CTGGGGCACATAGGGTGTT	
chCf1	GTCGAGGCAACGGTCAAAC	*chsC*
chCr1	TCGTAATGAGCAGCGAGACC	
Gel2f1	CGCATCCATCTTCAACGCAG	gel2
Gel2r1	TTGTAGGTGCTTTCGGGGTC	

Gel7f1	GGCACTGTAACTAGCTCCCC	*gel7*
Gel7r1	ACAGCAGAACCATACCAGCC	
Gel5f1	CTGTCACCTCCGCTACCAAG	*gel5*
Gel5r1	AAGAAGCAGACGAGCCTGAC	
RasAf1	CGAGCCGTCTCTCAACAAGA	*rasA*
RasAr1	AACCGGACGGATATGACGAC	
